# Supplementary material for: Nitrogen-plasma treated hafnium oxyhydroxide as an efficient acid-stable electrocatalyst for hydrogen evolution and oxidation reactions
Source: Nat Commun. 2019 Apr 4;10:1543. doi: 10.1038/s41467-019-09162-5 (PMC6449398; doi:10.1038/s41467-019-09162-5)
Supplement: Supplementary file 1 — Supplementary Information [file 41467_2019_9162_MOESM1_ESM.pdf]

Supplementary Information

Nitrogen-plasma treated hafnium oxyhydroxide as an efficient acid-stable electrocatalyst for hydrogen evolution and oxidation reactions

Yang *et al.*

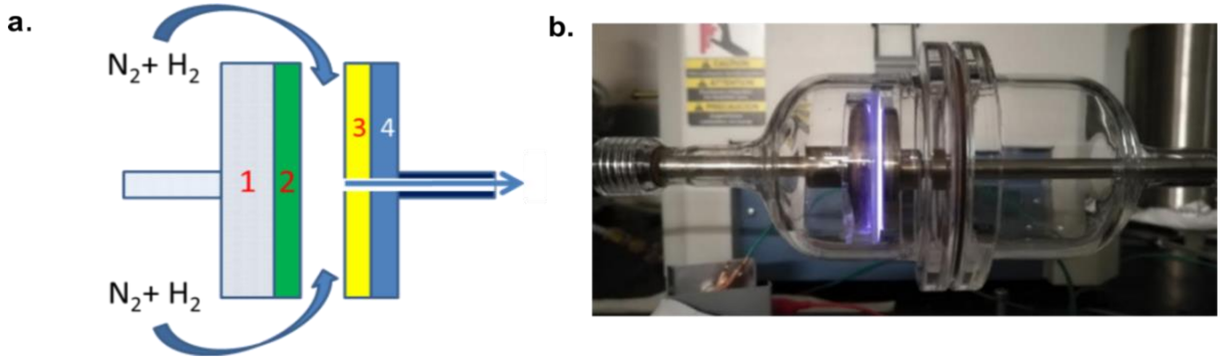

Supplementary Figure 1 Nitrogen plasma home-made dielectric barrier discharge (DBD) cell
a, Schematic drawing of the microplasma reactor. Electrode diameter was 1.5 in. for 1: HV electrode, 2. SiO_2 barrier, 3. Hf-coated Au substrates, and 4. Ground electrode. The temperature was monitored by a type K thermocouple attached to the ground electrode. **b,** Image during operation of the microplasma reactor. Stable plasma was formed in a 0.5 mm gap at 500 torr N_2 with a flow rate of 20 Nml min^{-1} and 15 W output from the AC supply.

Supplementary Note 1. Fabrication and physical characterization of Hf oxide thin films

The electrode samples of HfO_x were prepared by depositing a 20 nm thin film of Hf on a roughened gold substrate by Ar^+ sputtering deposition of Hf. The roughened Au was first prepared by electrochemically cycling process. The Hf thin film was quickly oxidized after exposure to air, and so to improve the electrical conductivity of the thin film electrode, N was implanted into Hf oxide by exposing the sample to a N_2 plasma generated in a home-built DBD plasma cell. The sample was heated by the plasma to roughly 100 ºC. Then the prepared thin film was characterized by HRXPS to determine the atomic concentration and oxidation states of the thin film. A one-hour exposure to the N_2 plasma typically results in atomic ration of N: Hf = 1:4 as determined by XPS. A representative HRXPS survey scan is shown in Supplementary Figure 2a. Surface morphologies of the electrode were measured by SEM, as shown in Supplementary Figure 2b, and deposition of the Hf oxide thin films and subsequent treatment in the N_2 plasma did not alter the appearance of the irregular shapes of Au nanoparticles, which looked similar to the roughened Au surfaces shown in Supplementary Note 9. Based on XPS, the HfO_xN_y film covered the exposed surface except for those regions that were shadowed by the particles during sputter deposition. The height of the Au particles is estimated to be 50-100 nm as measured by atomic force microscopy (AFM).

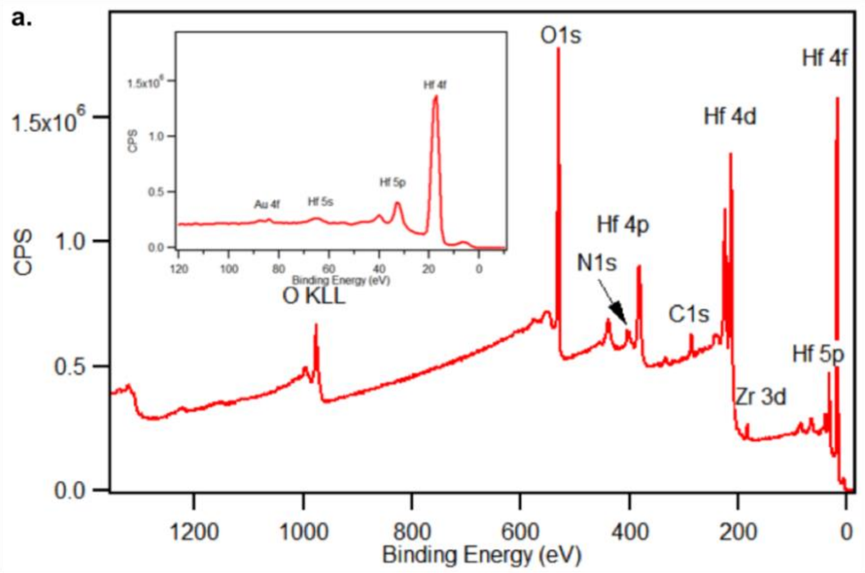

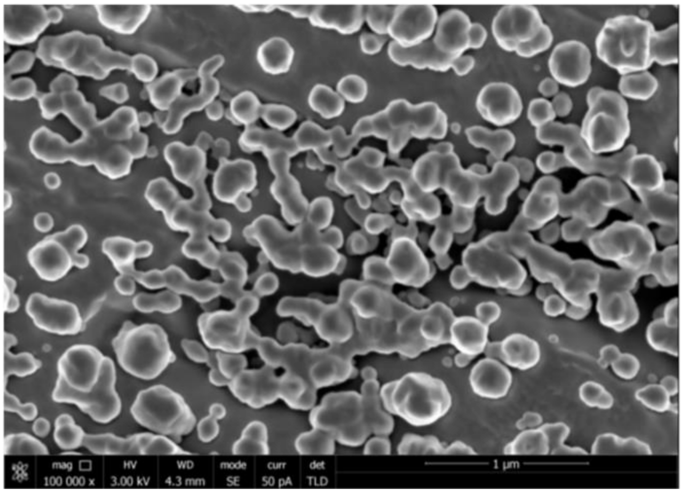

Supplementary Figure 2 Surface characterization by HRXPS and SEM
a, XPS survey scan of the N-modified Hf oxide thin film, indicating that the film nearly completely covered the Au substrate. A small amount (<3% relative to 100% Hf) Zr was present due to its presence as a common impurity in the Hf target. Inset: Magnification of the low binding energy region to show that there is no Pt contamination at 71 eV BE. **b**, SEM image of the electrode surface coated with the N-modified Hf oxide thin film.

Supplementary Note 2. Preparation of roughened Au substrates

An Au foil was cyclically scanned five times at 0.5 V s^{-1} in a 0.1 M KCl solution between -0.25 to 1.28 V vs. Ag/AgCl, followed by reduction at -0.25 V for 5 min. This treatment causes the surface to become rough with the surface area increased by 2.5 times. This treatment prevents the Hf oxide thin film from delaminating during H_2 generation in HER. Supplementary Figure 3 shows CV data from roughened and smooth Au foils in 0.1 M $HClO_4$.

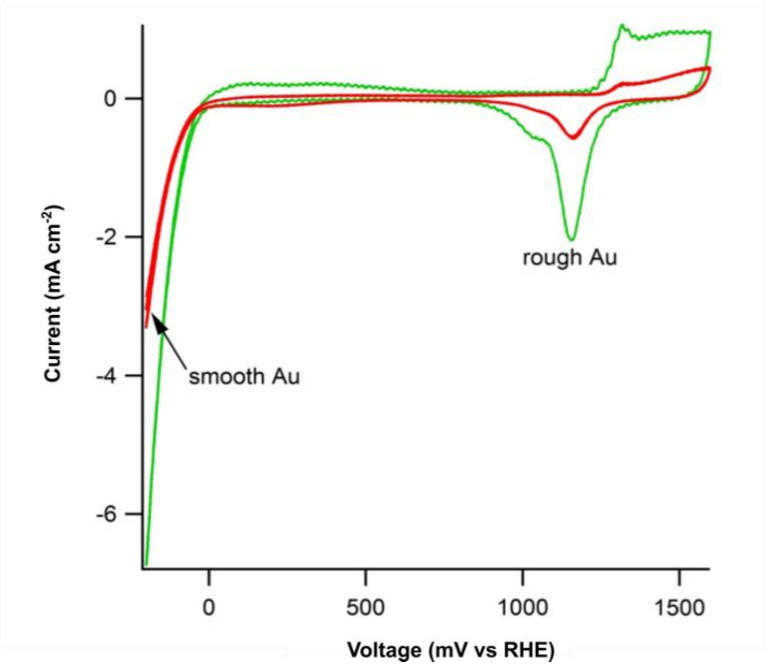

Supplementary Figure 3 Comparison of CV data of a smooth Au foil and a roughened Au foil in 0.1 M $HClO_4$ at 100 mV s^{-1} scan rate. The roughened sample shows twice the current in HER due to the increase of the surface area.

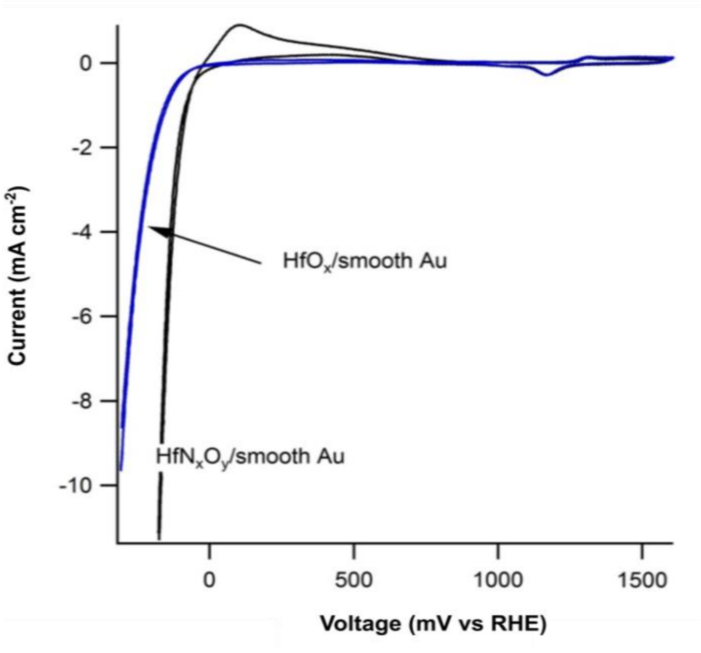

Supplementary Figure 4 Effect of N-modification of HfO_x thin films supported on smooth Au foil

Comparison of CV data of N-modified HfO_x and unmodified HfO_x thin films on smooth Au foils in 0.1 M $HClO_4$ at 100 mV s^{-1} scan rate. N-modification significantly increased the catalytic activity. This result again highlights the importance of N-modification, which is expected to increase the electrical conductivity.

Supplementary Note 3. Electrochemical surface area determined by double layer (DL) scans

The electrochemically active surface area (ECSA) was also estimated from the electrochemical double-layer capacitance of the catalytic surface. The electrochemical capacitance can be obtained from the scan-rate dependence of cyclic voltammetry (CV). Assuming no redox reaction, the capacitance is calculated from the capacitive current associated with double-layer charging/discharging according to the relation i= *v*C, where i is the current, *v* is the scan rate and C is the capacitance. The DL CV scans have been carried out for the gold foil, rough gold foil and the N-modified hafnium oxide in the potential range of 0.4 -0.5 V.

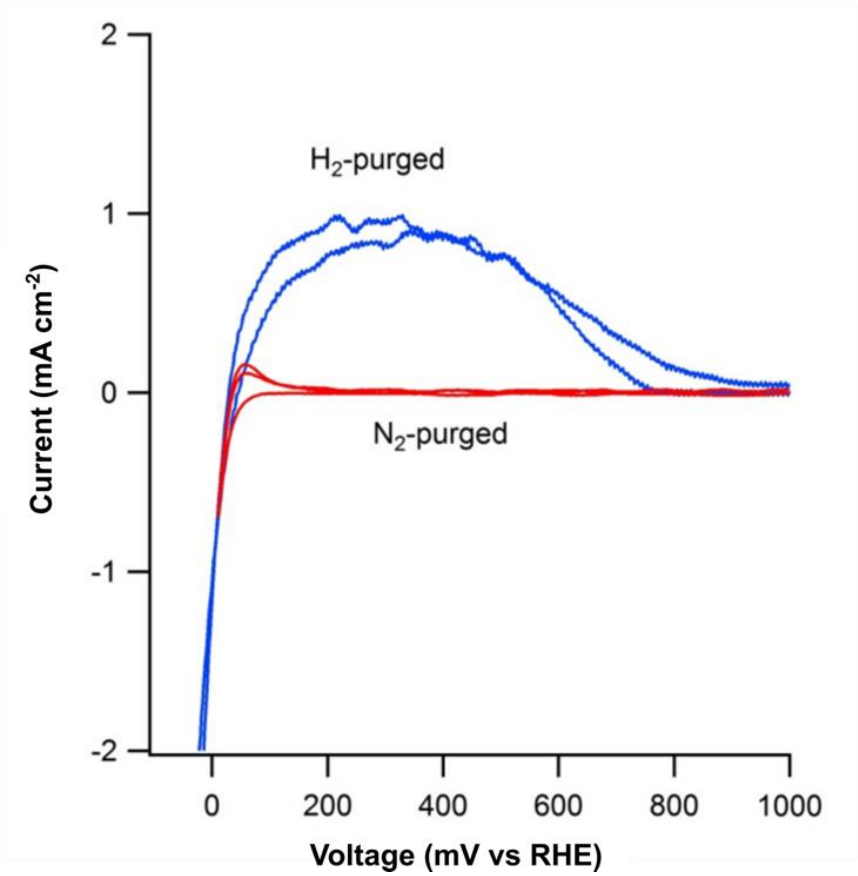

Supplementary Figure 5 Non-faradaic current from the double layer capacitance
Comparison of the slow-scan CV data of HfN_xO_y thin film on roughened Au in H_2- and N_2- purged 0.1 M $HClO_4$ at 5 mV s^{-1} scan rate.

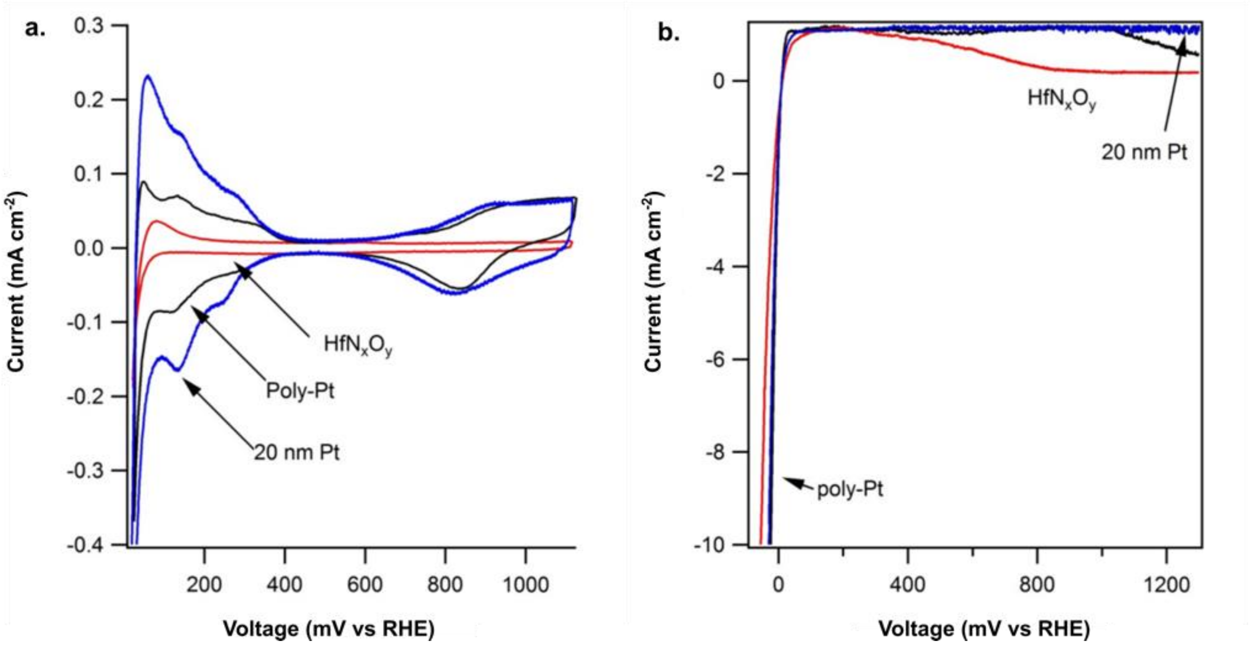

Supplementary Figure 6 CV data and HER/HOR of 20 nm Pt film, Pt foil, and HfN_xO_y film

a, CV data of N-modified HfO_x thin film (red), 20 nm Pt film on roughened Au (blue) and polycrystalline Pt (black) in N_2-purged 0.1 M $HClO_4$ at 20 mV/s scan rate. **b,** HER/HOR polarization scans for the samples shown in **a.** in H_2-purged 0.1 M $HClO_4$ at 5 mV/s scan rate. A 20 nm Pt thin film was deposited by Ar^+ ion sputter deposition on an Au foil that has been roughened. The CV data in N2-purged electrolyte and HER/HOR activity for polycrystalline Pt, a 20 nm Pt thin film deposited on roughened Au foil, and a HfN_xO_y thin film deposited on roughened Au foil are compared in Supplementary Figure 6a. The 20-nm Pt thin film shows twice larger H_{upd} peak area than the poly-Pt. There are no H_{upd} peaks for HfN_xO_y was observed. This CV comparison again excludes any HER-active PGM contamination in the HfO_xN_y thin film sample. Although the HER activity (normalized to the geometrical area) of the poly-Pt sample and 20 nm Pt thin film sample were similar to each other, we found that the HOR activity of the poly-Pt was higher than the 20 nm Pt thin film.

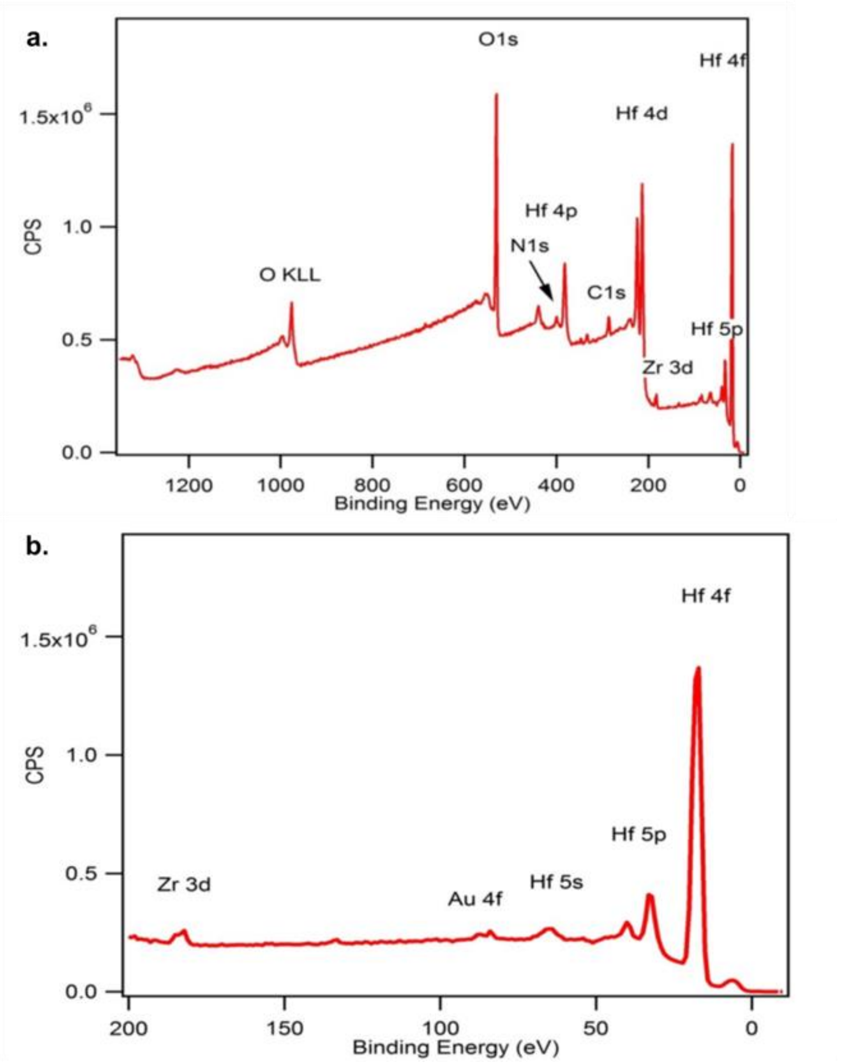

Supplementary Figure 7 HRXPS survey after electrochemical measurements

a, HRXPS survey scan of the N-modified HfOx thin film after electrochemical measurements (250 scans, -200 to 1600 mV). **b,** Zoom-in on the low binding energy region of the spectra. No Pt (71 eV BE) contamination was observed. This is important because Pt contamination could cause erroneous results that have been questioned regarding a report of highly active MoS_2. In our experimental method, we used a graphite counter electrode and we did not observe any trace Pt contamination on the thin film electrode.

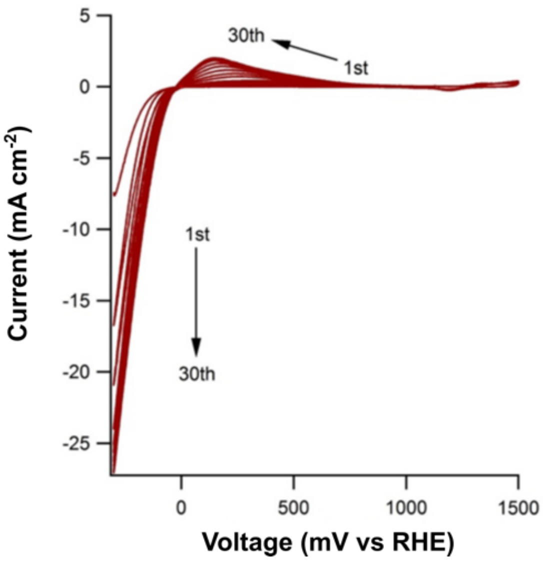

Supplementary Figure 8 Continuous CV scans in 0.1 M $HClO_4$ at 100 mV s^{-1} scan rate

The activity increased with the number of scans and approached a stable CV scan after 30 scans. Potential range: -300 to 1500 mV. The CV data are plotted using raw data without correction for the current-resistance loss. In the first 30 cycles, the CV data of the N-modified HfO_x thin film continuously increased in their anodic and cathode currents before reaching a stable state. These CV changes during the first 30 scans may be related to an electrochemical cleaning of the surface or activation process to form active hydroxide surface species.

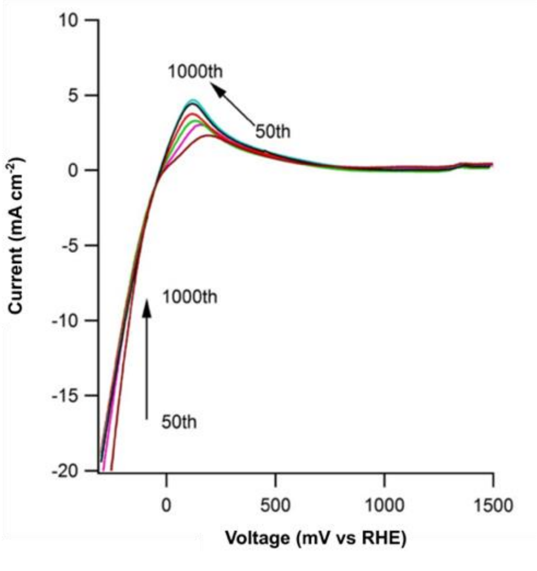

Supplementary Figure 9 Stability testing of a fresh sample by repeated CV scans

Positive scans from the 50th, 100th, 250th, 500th, 750th and 1000th CV scan are shown. In the HER region, the current density slightly drops after 100 scans. This could be caused by the increased H_2 generated and dissolved in the solution during increasing the time of CV scanning. Dissolved H_2 influences the zero potential on the RHE scale. On the other hand, increased H_2 in the solution causes a higher HOR current, and HOR currents were strongly influenced by the amount of H_2 gas dissolved in the solution. Conditions: N_2-purged 0.1 M $HClO_4$, 100 mV s^{-1}. Potential range: -300 to 1500 mV. The CV data are plotted using raw data without correction for the current-resistance loss.

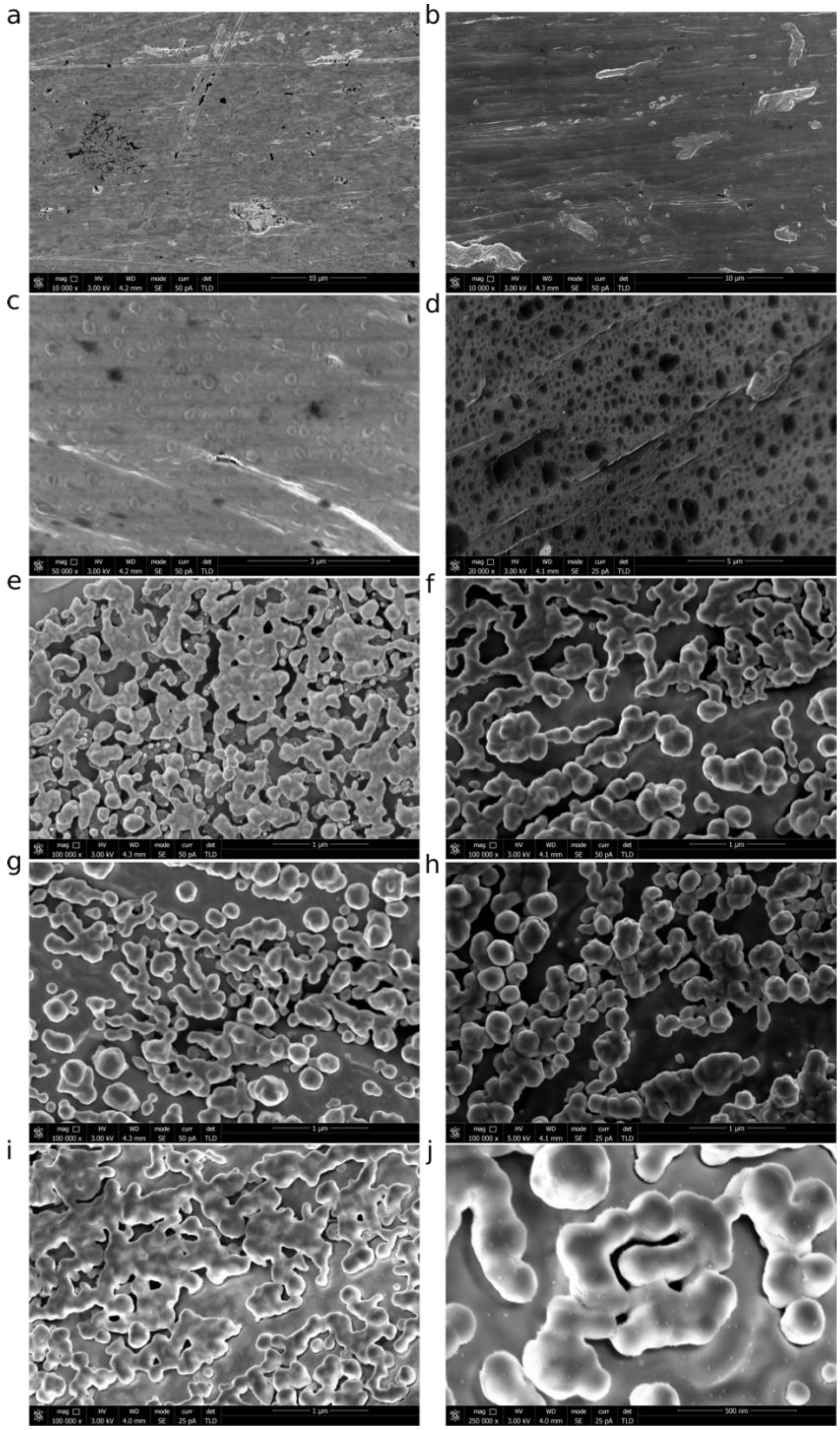

Supplementary Figure 10 Morphologies of electrodes measured by SEM

SEM images are shown the surface morphologies of different electrodes. **a**, smooth Au foil; **b**, 20 nm HfO_x coated on smooth Au foil; **c**, N-modified 20 nm HfO_x on smooth Au foil; **d**, after 250 CV cycles of **c** (stage tilted 45°); **e**, roughened Au foil; **f**, 20 nm HfO_x coated on roughened Au foil; **g**, N-modified HfO_x on roughened Au foil; **h**, after 250 CV cycles of **f** (stage tilted 45°); **i**, **j,** after 250 CV cycles of **g** (stage tilted 45°, **i** with 1 µm scale bar, and with 500 nm scale bar). The CV scan conditions for **d**, **h**, **i** and **j**: 0.1 M $HClO_4$, -200-1600 mV, 100 mV s^{-1}, 250 cycles.

References

1. Mortari A, Maaroof A, Martin D and Cortie MB (2007) Mesoporous gold electrodes for sensors based on electrochemical double layer capacitance. *Sensors and Actuators B: Chemical* 123: 262-268.
